# Prognostic analysis of neovascular glaucoma treatment in 227 patients: A retrospective observational study

**DOI:** 10.1097/MD.0000000000045192

**Published:** 2025-10-10

**Authors:** Xinhong Jiang, Xizi Cheng, Guoteng Chai, Weiyi Zhang, Hongming Liu, Qiqi Shen, Yuling Zou

**Affiliations:** a The Affiliated Eye Hospital, Jiangxi Medical College, Nanchang University, Jiangxi Province Key Laboratory of Ophthalmology and Vision Sciences, Jiangxi Clinical Research Center for Ophthalmic Disease, Jiangxi Provincial Key Laboratory of Vitreoretinal Diseases for Health, Nanchang, Jiangxi, China.

**Keywords:** anti-glaucoma surgery, neovascular glaucoma, treatment, vitrectomy

## Abstract

To investigate the etiology, therapies, and outcomes of neovascular glaucoma, guiding prevention and treatment strategies. This study retrospectively analyzed the causes, treatment methods, and efficacy in 218 hospitalized patients (227 eyes) diagnosed with neovascular glaucoma at the Affiliated Eye Hospital of Nanchang University from 2017 to 2024. In this study, diabetic retinopathy occurred in 93 eyes (41.0%) and retinal vein occlusion in 52 eyes (22.9%), collectively representing 134 eyes (59.0%) with these primary pathologies. No light perception to 0.02 vision was observed in 192 eyes (86.1%) before treatment and 191 eyes (85.6%) posttreatment, with no significant difference (*P* > .05). Elevated intraocular pressure (>21 mm Hg) declined from 94.0% (213 eyes) pretreatment to 41.7% (93 eyes) posttreatment, with 50.2% (112 eyes) achieving 10–21 mm Hg (*P* < .05). Significant intraocular pressure reduction occurred in vitrectomy cases, combined vitrectomy/anti-glaucoma surgery, standalone anti-glaucoma procedures, and anti-vascular endothelial growth factor therapies (all *P* < .05), whereas conservative treatment showed no significant effect. Neovascular glaucoma (NVG) is a type of secondary refractory glaucoma predominantly caused by conditions such as diabetic retinopathy and retinal vein occlusion. Standard treatments include vitrectomy, anti-glaucoma surgery, and anti-vascular endothelial growth factor therapy, commonly tailored and combined according to the specific needs of the patient. Although these aggressive treatments can effectively manage intraocular pressure in Neovascular glaucoma (NVG), improving vision remains challenging.

## 1. Introduction

Neovascular glaucoma (NVG) is a severe and challenging form of glaucoma, characterized by neovascularization of the iris (NVI) and the anterior chamber angle (NVA). These developments can lead to angle closure and sustained increased intraocular pressure, typically following conditions such as diabetic retinopathy (DR), retinal vein occlusion (RVO), previous retinal detachment, and ocular tumors.

Glaucomatous optic neuropathy arises primarily from inadequate ocular blood flow due to elevated intraocular pressure or other risk factors,^[1]^ with impaired retinal microvascular perfusion and subsequent dysfunction in vascular autoregulation playing crucial roles.^[2]^ Consequently, ocular microcirculatory perfusion significantly contributes to understanding the pathogenesis of glaucoma.^[3]^Neovascular glaucoma (NVG) predominantly associates with vascular conditions that lead to ocular hypoxia, as commonly observed in DR, RVO, and ocular ischemic syndrome (OIS), and also occurs in conditions like uveitis and ocular tumors.^[4]^

Studies indicate that patients with neovascular glaucoma mainly suffer from DR and RVO, which represent 40.9% and 22.9% of cases respectively, aligning with previous findings.^[5,6]^Diabetic patients globally account for more than 30% of NVG cases.^[7]^in this study, 41.0% of the patients were those with DR,probably associated with the rising incidence of diabetes in China. Earlier research shows that within 5 years of being diagnosed with type 2 diabetes, 1.74% of patients develop proliferative DR (PDR), and 0.14% develop NVG.^[8]^In China, the contributions of DR, RVO, and OIS to NVG cases are 39.7%, 22.9%, and 2.3%,^[7]^ respectively. In cases of central RVO (CRVO), ocular neovascularization is a complication seen in ischemic CRVO but not in nonischemic CRVO.^[9]^

Despite advances in treatments like vitrectomy, panretinal laser photocoagulation, and anti - vascular endothelial growth factor (VEGF) therapies, managing NVG remains difficult.^[4]^ Treatment options are varied, but the prognosis often remains poor, significantly affecting patients’ quality of life and vision.^[10]^

Timely identification and intervention are crucial for postponing the advancement of neovascular glaucoma (NVG) and preventing irreversible vision loss. Once NVG is established and intraocular pressure (IOP) is elevated, the primary objective shifts to controlling the increased IOP rather than solely addressing the underlying disease causing NVG. For patients with NVG, alongside rigorous management of the primary disease, anti-VEGF therapy can facilitate neovascularization regression, filtering surgery can help maintain eye pressure control, and retinal photocoagulation can mitigate retinal hypoxia induced by the primary condition. These therapeutic strategies collectively constitute the standard management practice to delay NVG progression and associated vision loss.^[7]^ A comprehensive evaluation of the anterior segment is crucial for early detection of neovascularization on the iris surface or in the anterior chamber angle, enabling early diagnosis of NVG and prompt therapeutic intervention. Approximately 60% of patients with ischemic CRVO develop anterior segment neovascularization within weeks to 2 years post-onset,^[10]^ and the incidence of neovascularization of the iris (NVI) in PDR can reach up to 65%, with a 33% risk of contralateral eye NVG in patients with unilateral NVG due to diabetes.^[10]^

Significant knowledge gaps persist in the NVG literature. These include regional variations and incomplete understanding of the etiological spectrum, a lack of consensus on the long-term efficacy and optimal combination approaches for treatments such as anti-VEGF therapy versus trabeculectomy, and a critical need for effective strategies to improve visual function. To address these gaps, this retrospective study analyzed data from 218 NVG patients (227 eyes) at the Eye Hospital of Nanchang University. The findings specifically contribute by: establishing diabetes retinopathy (DR) and RVO as the predominant etiologies within this Chinese cohort; demonstrating the efficacy of vitrectomy combined with anti-glaucoma surgery and adjunctive anti-VEGF therapy in achieving intraocular pressure control; and highlighting the substantial challenge that visual improvement continues to pose despite effective pressure reduction.

## 2. Materials and methods

### 2.1. Research subjects

This study collected data from 218 patients (227 eyes) diagnosed with neovascular glaucoma at the Affiliated Eye Hospital of Nanchang University from May 2017 to May 7, 2024. The clinical characteristics of these patients were statistically analyzed, including gender, age, affected eye, treatment methods, visual acuity, intraocular pressure before treatment and at the last follow-up, lens status, postoperative complications, previous surgical history, and history of other diseases such as hypertension and diabetes.

### 2.2. Ethics statement

The study was conducted in accordance with the Declaration of Helsinki, and approved by the Medical Research Ethics Committee of the Affiliated Eye Hospital of Nanchang University (protocol code YLP20250053).

### 2.3. Diagnostic criteria and exclusion criteria

Diagnostic Criteria: Iris neovascularization and/or trabecular vascular tissue proliferation^[10]^;Inclusion Criteria: (1) Diagnosed with neovascular glaucoma. Exclusion Criteria: (1) Presence of other types of glaucoma; (2) Incomplete clinical data.

### 2.4. Research methods

This was a retrospective observational study. This study gathered data through a comprehensive review of electronic medical records of patients diagnosed with neovascular glaucoma at the Affiliated Eye Hospital of Nanchang University from May 2017 to May 7, 2024 on the sex, age, eye type, treatment methods, visual acuity, intraocular pressure, and histories of other diseases, such as hypertension and diabetes, for all patients (refer to Table 1). It analyzed the primary diseases causing neovascular glaucoma (NVG), treatment approaches, and patient prognoses. Visual acuity categories were defined as no light perception, light perception ~ 0.02, 0.02 ~0.05, and >0.05. intraocular pressure was categorized into 3 groups: IOP<10mmHg、10mmHg ≤ IOP ≤ 21mmHg、IOP>21mmHg

**Table 1:** General information.

		N/%
Sex	Male	154 (67.8%)
	Female	73 (32.2%)
Age	–	58.37 ± 15.54
Eye category	Oculus dexter	102 (44.9%)
	Oculus sinister	125 (55.1%)
Condition of the lens	Natural lens	158 (69.8%)
	Intraocular lens eye	61 (26.9%)
	Aphakia	6 (2.6%)
	Dislocation of the lens	2 (0.9%)
Postoperative complications	KP*	2 (6.7%)
	Tyndall effect (+)	1 (3.3%)
	Shallow anterior chamber	5 (16.7%)
	Blood in the anterior chamber	10 (33.3%)
	Absence of the lens	5 (16.7%)
	Intraocular pressure is low	7 (23.3%)
History of eye surgery	Yes	81 (35.7%)
	No	146 (64.3%)
Other medical history	Hypertension	83 (36.6%)
	Diabetes	76 (33.5%)

* KP:Keratic Precipitates.

### 2.5. Treatment methods

Depending on the patient’s condition, various treatment methods were employed, categorized into: vitrectomy, combined vitrectomy with anti-glaucoma surgery, anti-glaucoma surgery alone, anti-VEGF treatment, and conservative treatment. In cases with preoperative concurrent vitreous hemorrhage or tractional retinal detachment, vitrectomy was performed on 58 eyes (25.6%), and combined vitrectomy with anti-glaucoma surgery was performed on 17 eyes (7.5%). Additionally, if preoperative conditions necessitate cataract surgery to improve the surgeon’s visual field, cataract extraction was conducted, with the decision to implant an artificial lens made based on the patient’s ocular status. Retinal laser photocoagulation was also administered. The selection of a vitreous cavity filler, such as silicone oil or gas, depended on the underlying disease. Anti-glaucoma procedures included filtering surgeries, like trabeculectomy and peripheral iridectomy, as well as the implantation of GDDs. (e.g., drainage implants or valves), and destructive surgeries on the ciliary body, which comprise cyclo-cryotherapy, cyclophotocoagulation, and cyclodestructive ultrasound surgery, among others.

### 2.6. Statistical methods

The statistical evaluation of the data was performed utilizing SPSS 26.0 software. The measurement data are expressed as mean ± standard deviation (x ± s) and were subjected to analysis through nonparametric testing methods. Count data were expressed as percentages (%) and were analyzed utilizing either χ² tests or Fisher exact test. The threshold for statistical significance was established at α = 0.05.

## 3. Results

### 3.1. Etiology analysis

A total of 218 patients with neovascular glaucoma (227 eyes) were examined, comprising 154 males (67.84%) and 73 females (32.16%), with an average age of 58.37 ± 15.54 years. The onset was unilateral in 209 cases and bilateral in 9 cases, all of which were attributed to DR. The predominant cause of NVG was identified as DR in 93 eyes (41.0%), followed by RVO in 52 eyes (22.9%), prior retinal detachment in 17 eyes (7.5%), retinal vasculitis in 6 eyes (2.6%), uveitis in 5 eyes (2.2%), retinal artery occlusion in 2 eyes (0.9%), OIS in 2 eyes (0.9%), trauma in 1 eye (0.4%), and intraocular tumor in 1 eye (0.4%). The etiology remained undetermined in the remaining 48 eyes. Additionally, preoperative conditions included hyphema in 28 eyes (12.3%), vitreous hemorrhage in 61 eyes (26.9%), and associated retinal detachment in 28 eyes (12.3%).

### 3.2. Management of neovascular glaucoma

Surgical interventions for glaucoma were carried out on a total of 102 eyes (44.9%) to manage intraocular pressure. These interventions included trabeculectomy in 6 eyes (2.6%), peripheral iridectomy in 9 eyes (4.0%), drainage valve or implant surgeries in 24 eyes (10.6%), cycloablation in 48 eyes (21.2%), cyclophotocoagulation in 9 eyes (4.0%), and ultrasound cyclocoagulation in 9 eyes (4.0%). Additionally, 29 eyes received concomitant anti-VEGF treatment. Alone, anti-VEGF treatment was administered to 35 eyes (15.4%). Meanwhile, 11 patients opted exclusively for retinal laser coagulation and conservative management with eye drops to reduce intraocular pressure. Four cases, each without light perception, underwent enucleation or evisceration due to severe discomfort from elevated intraocular pressure. All patients were treated with topical intraocular pressure-lowering medication preoperatively.

### 3.3. Prognostic analysis of treatment in patients with neovascular glaucoma

Overall, the proportion of eyes with visual acuity ranging from no light perception to 0.02 was 192 (86.1%) preoperatively and 191 (85.6%) postoperatively, showing no statistically significant change (*P* > .05). The detailed distribution of visual outcomes for this specific acuity range, stratified by treatment modality, is comprehensively presented in Table 2.

**Table 2:** Distribution of vision in NVG patients before and after treatment.

Treatment mode	Time	No light perception	Light perception −0.02	>0.02–0.05	>0.05	*P*
PPV	Before treatment	20 (34.5%)	31 (53.4%)	1 (1.7%)	6 (10.3%)	.585
	After treatment	24 (41.4%)	27 (46.6%)	3 (5.2%)	4 (6.9%)	
Combined vitrectomy with anti-glaucoma surgery	Before treatment	1 (5.9%)	13 (76.5%)	1 (5.9%)	2 (11.8%)	.287
	After treatment	4 (23.5%)	12 (70.6%)	0 (0.0%)	1 (5.9%)	
Anti-glaucoma surgery alone	Before treatment	50 (49.0%)	42 (41.2%)	1 (1.0%)	9 (8.8%)	.787
	After treatment	51 (50.0%)	38 (37.3%)	3 (2.9%)	10 (9.8%)	
Anti-VEGF treatment	Before treatment	8 (22.9%)	17 (48.6%)	2 (5.7%)	8 (22.9%)	.962
	After treatment	9 (25.7%)	15 (42.9%)	2 (5.7%)	9 (25.7%)	
Conservative treatment	Before treatment	5 (45.5%)	6 (54.5%)	0 (0.0%)	0 (0.0%)	1
	After treatment	5 (45.5%)	6 (54.5%)	0 (0.0%)	0 (0.0%)	
Total	Before treatment	84 (37.7%)	109 (48.9%)	5 (2.2%)	25 (11.2%)	.625
	After treatment	93 (41.7%)	98 (43.9%)	8 (3.6%)	24 (10.8%)	

NVG = neovascular glaucoma, PPV = pars plana vitrectomy.

Preoperatively, 212 eyes (95.0%) had an initial intraocular pressure (IOP) exceeding 21 mm Hg. Posttreatment, IOP remained above 21 mm Hg in 93 eyes (41.7%), while 112 eyes (50.2%) achieved IOP between 10 mm Hg and 21 mm Hg, showing a statistically significant reduction in IOP (*P* < .05).. Reporting of aggregate preoperative and postoperative counts of eyes achieving an IOP between 10 and 21 mm Hg across different treatment modalities is provided in the text, with detailed IOP distributions stratified by therapeutic approach presented in Table 3.

**Table 3:** Distribution of intraocular pressure before and after treatment in NVG patients.

Treatment mode	Time	<10	10–21	>21	*P*
PPV	Before treatment	0 (0.0%)	4 (6.9%)	54 (93.1%)	.000
	After treatment	5/(8.6%)	29 (50.5%)	24 (41.4%)	
combined vitrectomy with anti-glaucoma surgery	Before treatment	0 (0.0%)	0 (0.0%)	17 (100%)	.000
	After treatment	3 (17.6%)	9 (52.9%)	5 (29.4%)	
anti-glaucoma surgery alone	Before treatment	0 (0.0%)	1 (1.0%)	101 (99%)	.000
	after treatment	8 (7.8%)	55 (53.9%)	39 (38.2%)	
anti-VEGF treatment	Before treatment	0 (0.0%)	5 (14.3%)	30 (85.7%)	.002
Conservative treatment	Before treatment	0 (0.0%)	0 (0.0%)	11 (100%)	.090
	After treatment	0 (0.0%)	4 (36.4%)	7 (63.6%)	
Total	Before treatment	0 (0.0%)	10 (4.5%)	213 (95.5%)	.000
	After treatment	18 (8.1%)	112 (50.2%)	93 (41.7%)	

NVG = neovascular glaucoma, PPV = pars plana vitrectomy, VEGF = vascular endothelial growth factor.

The unit for intraocular pressure is expressed in mm Hg;NVG:neovascular glaucoma.

There were statistically significant differences in intraocular pressure (IOP) following vitrectomy, combined vitrectomy with anti-glaucoma surgery, anti-glaucoma surgery alone, and anti-VEGF (anti-VEGF) therapy (*P* < .05). Preoperatively, the number of eyes with IOP > 21 mm Hg was 54 (93.1%), 17 (100%), 101 (99.0%), and 29 (82.9%) in the respective treatment groups. Postoperatively, the number of eyes achieving IOP within the range of 10 mm Hg ≤ IOP ≤ 21 mm Hg was 29 (50.0%), 9 (52.9%), 55 (53.9%), and 15 (42.9%), respectively. Among the 11 eyes receiving conservative treatment, 100% (11 eyes) exhibited IOP exceeding 21 mm Hg preoperatively. Posttreatment, only 4 eyes (36.4%) achieved IOP between 10 mm Hg and 21 mm Hg, while the remaining 7 eyes (63.6%) maintained IOP above 21 mm Hg, showing no statistically significant difference (*P* > .05).

## 4. Discussion

The treatment of neovascular glaucoma (NVG) primarily targets the formation of new blood vessels and the elevation of intraocular pressure. Presently, visual function can be optimally preserved through various approaches including medication, anti-VEGF therapy, surgical intervention, retinal photocoagulation (PRP), and management of the primary disease.^[11]^ Research has established inflammation as a crucial factor in NVG pathogenesis,^[12]^ with increased levels of cytokines such as interleukin (IL-6, IL-8, IL-10), VEGF, and monocyte chemoattractant protein-1 (MCP-1) in the aqueous humor of affected eyes.^[13]^ Importantly, transforming growth factor-β (TGF-β) may play a significant role in NVG progression.^[14]^ VEGF is the most extensively studied pro-angiogenic factor in NVG. Studies indicate that VEGF and VEGF-mRNA levels are significantly elevated in ischemic retinas,^[15]^ whereas anti-VEGF drugs can induce regression of iris neovascularization, thus improving conditions for further surgical intervention. However, The impact of anti-VEGF medications generally last between 4 to 6 weeks, necessitating additional treatments as iris neovascularization may recur. The contraction of the fibrovascular membrane on the iris and trabecular meshwork surface leads to peripheral iris anterior synechiae, compromising the aqueous humor outflow pathway and causing sustained intraocular pressure increases. A combined approach of anti-VEGF therapy and trabeculectomy can be considered; however, challenges such as postoperative scarring, conjunctival inflammation, and new vascular membranes obstructing the filtration pathway, along with vigorous neovascular regrowth post-surgery, contribute to a high failure rate of NVG trabeculectomy. Furthermore, postoperative anterior chamber hemorrhage may also result in trabeculectomy failure.^[16]^

In this study, a total of 75 eyes (33.04%) underwent vitrectomy alone or combined with glaucoma surgery, including 36 eyes with vitreous hemorrhage and 2 (2.67%) with tractional retinal detachment. The procedures resulted in small incisions, effective clearance of hemorrhage, and significant retinal reattachment, thereby substantially improving postoperative vision.A previous meta-analysis identified the primary risk factors for neovascular glaucoma (NVG) following pars plana vitrectomy (PPV) in patients with PDR as elevated baseline intraocular pressure, preoperative iris neovascularization, simultaneous cataract surgery, and postoperative vitreous hemorrhage.^[17]^ Destructive ciliary body surgeries, such as ciliary body cryotherapy, photocoagulation, and ultrasound ciliary body plasty, have distinct roles. Cryotherapy, though invasive with substantial tissue damage and high risk of complications, effectively reduces intraocular pressure by causing ciliary body atrophy and decreased aqueous humor production. Meanwhile, ultrasound ciliary body plasty (UCP) is gaining acceptance in clinical practice and has proven safe and effective for glaucoma treatment.^[18]^ UCP is noninvasive applied to the ciliary process that produces aqueous humor to perform accurate, controllable and gentle thermocoagulation, while maintaining the blood-aqueous humor barrier, melting the ciliary epithelial cell layer, reducing the production of aqueous humor, opening the scleral pathway, increasing the outflow of aqueous humor, achieving a double reduction of IOP, and alleviating eye pain.^[19]^Recent innovations in drainage pathways include the posterior aqueous drainage procedure, with posterior glaucoma implants protecting the corneal endothelium from surgical damage and significantly reducing intraocular pressure in patients with refractory glaucoma. Ahmed glaucoma valve implantationis a safe and effective surgical method to treat the uncontrolled increase in intraocular pressure after PPV,Sevgi Subasi showed that patients with NVG and secondary glaucoma after PPV had surgery success rates of 96.2% and 87.5% at 6 months and 12 months, respectively, and that both groups were safe and effective in terms of IOP, BCVA, and complications.^[20]^Unlike Ahmed glaucoma drainage valves, Ex-PRESS drainage implants are smaller and offer distinct benefits and limitations for different patient profiles. Panretinal photocoagulation (PRP) is another therapeutic option that delays NVG progression,^[21]^ with an adequate number of laser points serving as a protective factor. For NVG patients, PRP should be performed promptly once intraocular pressure is reduced and corneal clarity is achieved.

The study encompassed diverse therapeutic interventions including PPV, combined PPV with anti-glaucoma procedures, standalone anti-glaucoma surgery, anti-VEGF therapy, and conservative management. This multimodal approach demonstrates notable variations when compared with other contemporary studies. Specifically, Liao et al. (2016) primarily utilized glaucoma drainage device (GDD) implantation (103 eyes) and transscleral cyclophotocoagulation (TSCPC) (22 eyes), establishing GDD as the preferred intervention for intraocular pressure control in NVG patients with residual visual potential.^[22]^ In contrast, the study did not predominantly favor any single treatment modality. Furthermore, AlRubaie et al. (2021) reported that 335 eyes (56.1%) underwent panretinal photocoagulation (PRP), while 459 eyes (77%) achieved IOP control through either pharmacological or surgical interventions.^[6]^ Although PRP was not emphasized in the study, it provided detailed analyses of IOP outcomes across various surgical and medical treatments. Similarly, Malgi et al. (2021) documented extensive application of multiple therapies including TSCPC, glaucoma surgery, anti-VEGF agents, PRP, and vitreoretinal surgery.^[23]^ While the study similarly reported visual and tonometric outcomes for multiple interventions, distinct proportional distributions and analytical emphases were observed. These disparities in treatment preferences primarily stem from geographical, temporal, demographic, and research focus variations across studies

Limitations of this study include: The small sample size and generally unsatisfactory outcomes for NVG patients hamper the ability to achieve favorable prognoses with a single treatment modality, precluding separate reporting of outcomes for each intervention. Being a hospital-based study, conducted in a large specialized eye hospital, introduces selection bias associated with the source of the patients.

This study provides significant contributions to the field of ophthalmology by offering a comprehensive analysis of the etiology of neovascular glaucoma (NVG), which confirms DR and RVO as its primary pathogenic factors, thereby supplying critical evidence for early diagnosis and prevention. Furthermore, it delivers a detailed evaluation of multiple treatment modalities – including vitrectomy, anti-glaucoma surgery, and anti-VEGF (anti-VEGF) therapy – and demonstrates that although active treatment can effectively control intraocular pressure in NVG cases, visual improvement remains challenging, thus offering valuable guidance for clinical decision-making.To enhance the validity and generalizability of these findings, future studies should aim to enlarge the sample size to mitigate limitations and allow more precise assessment of long-term treatment efficacy and safety. Additionally, further investigation into the role of inflammatory factors in the pathogenesis of NVG may reveal novel therapeutic targets.In clinical terms, this work underscores the importance of early identification and intervention in NVG, assisting clinicians in devising individualized treatment strategies that effectively regulate intraocular pressure. Although restoring visual acuity remains difficult, this study outlines a meaningful direction for improving patients’ quality of life and visual function.

## Author contributions

**Conceptualization:** Xinhong Jiang, Xizi Cheng.

**Data curation:** Guoteng Chai, Weiyi Zhang.

**Formal analysis:** Xinhong Jiang, Xizi Cheng.

**Funding acquisition:** Yuling Zou.

**Investigation:** Xinhong Jiang, Xizi Cheng, Qiqi Shen.

**Methodology:** Xinhong Jiang, Xizi Cheng.

**Project administration:** Yuling Zou.

**Resources:** Yuling Zou.

**Software:** Guoteng Chai.

**Supervision:** Yuling Zou.

**Validation:** Weiyi Zhang, Hongming Liu.

**Visualization:** Qiqi Shen.

**Writing – original draft:** Xinhong Jiang, Xizi Cheng.

**Writing – review & editing:** Yuling Zou.
